# Charge Carrier and Spin Diffusion in a Polycrystalline and Single Crystal Lead Halide Perovskite Semiconductor

**DOI:** 10.1021/acsphotonics.6c00657

**Published:** 2026-06-29

**Authors:** Kazimieras Nomeika, Justina Jovaišaitė, Ramu̅nas Aleksieju̅nas, Xinwen Zhang, Duong Nguyen Minh, Md Azimul Haque, Matthew C. Beard, Joseph M. Luther, Justin C. Johnson

**Affiliations:** † Light Conversion Ltd., Keramiku̧ str. 2B, LT-10233 Vilnius, Lithuania; ‡ Faculty of Physics, Institute of Photonics and Nanotechnology, 54694 Vilnius University, Saulėtekis Ave. 3, LT-10257 Vilnius, Lithuania; § 53405 National Laboratory of the Rockies, 15013 Denver West Pkwy, Golden, Colorado 80401, United States; ∥ RASEI, A Joint CU-Boulder/NLR Energy Institute, Boulder, Colorado 80309, United States

**Keywords:** transient grating, perovskite, semiconductor, spin, diffusion

## Abstract

The directed movement of spin-bearing excitations in semiconductors can enable many potential schemes for spintronic applications. Understanding the mechanism of spin motion, as opposed to charge carrier or exciton motion, may require specialized techniques and sample conditions. Here we employ the noncontact and time-resolved technique of light-induced transient grating spectroscopy (LITG) to measure both carrier and spin motion in a perovskite semiconductor with varying crystallinity. The carrier motion aligns with expectations from past studies undertaken at low fluence, revealing an ambipolar diffusion coefficient on the order of 1 cm^2^/sec and diffusion length of roughly 1.5 μm for single crystals that is strongly attenuated as crystallite size decreases. The spin diffusion is measured with cross-polarized excitation beams and uncovers an intensity-dependent coefficient rising above 100 cm^2^/sec. The fast room-temperature spin relaxation limits the spin diffusion length, but the remarkable speedup of spin over carrier diffusion suggests a mechanism involving a combination of exchange-mediated and doping effects that enhance spin transport.

## Graphical abstract

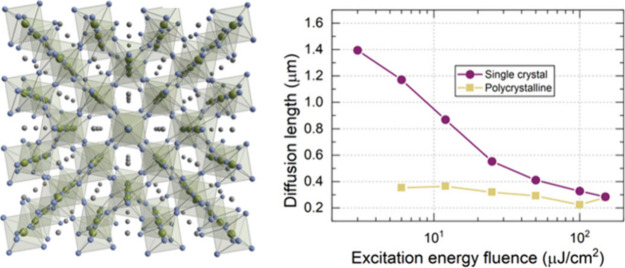

## Introduction

Diffusion of excitons, charge carriers, and their associated spins represents an important process that enables energy transduction, spin-related computing, and photocatalysis schemes.^1^ In models for conventional diffusion, two important parameters must be determined to characterize the time-dependent trajectory of motion: the population/spin relaxation time and the diffusion coefficient.^2^ Population relaxation can be measured routinely with a variety of techniques, such as time-resolved photoluminescence or transient absorption. Common techniques to measure spin relaxation include circularly polarized pump–probe or luminescence spectroscopy^3,4^ and Faraday or Kerr rotation experiments.^5–10^ Measurements of diffusion are often performed in a distinct mode compared with population relaxation measurements, including the use of the aforementioned spectroscopies adapted to microscopy methods^11^ or utilizing device-like architectures.^12–14^ The disparities between conditions employed, including pump fluence, magnetic field strengths, and time scales probed, can make it difficult to arrive at a full dynamical picture of the exciton and charge population and spin dynamics.

In the present investigation we demonstrate a lesser-used but powerful technique termed light-induced transient grating (LITG) spectroscopy, sometimes also referred to as four-wave mixing.^15^ Initially, LITG was developed as a tool for investigation of charge carrier dynamics in molecular crystals^13^ and inorganic semiconductors.^16,17^ Despite early origins and many applications, LITG has harbored the reputation of a more complex alternative to other ultrafast techniques that require magnetic fields, high spatial resolution, or low temperatures for characterizing diffusion. Recent user-friendly adaptations have improved accessibility and enable streamlined and comprehensive investigation. In addition to other applications, LITG was successfully employed to investigate carrier diffusion and recombination over a wide range of carrier densities in metal–halide perovskites.^18–20^ Our prior work using LITG determined diffusion lengths in polycrystalline methylammonium lead iodide perovskite thin films, concluding that high barriers to carrier diffusion exist at grain boundaries.^21^

Most often, LITG is used to observe carrier or exciton transport when a transient diffraction grating is created from an interference pattern of two coherent pulses with linear parallel polarization. However, it has been demonstrated by Cameron et al. that spin diffusion can also be observed in LITG, if cross-polarized pump pulses are used for grating recording.^22^ This technique has been first confirmed in GaAs quantum wells,^23,24^ and later in more complex bulk and nanoscale systems that typically have ps-scale relaxation process related to phonon interactions, spin–orbit coupling, and exchange.^25,26^ In systems with spin-oriented excitons, cross-polarized LITG tracks the spin motion and its fine structure relaxation,^27,28^ unlike techniques like time-resolved Faraday rotation, which probe net spin density. Depending on the targeted optoelectronic properties, selective probing of the dynamics of exciton angular momentum is an important feature of LITG. An additional advantage of LITG is its zero-signal background, arising from the phase matching associated with noncollinear four-wave mixing. High signal-to-noise measurements and thus accurate spin relaxation and diffusion are achievable in one simple experimental setup without magnetic fields, microscope objectives, or specialized detection schemes.

Here we obtain LITG results for both population and spin grating measurements, which simultaneously and separately probe dynamics associated with population and spin relaxation and their attendant diffusion. We find that spin diffusion occurs on a time scale 2 orders of magnitude faster than population diffusion at room temperature for a prototypical bulk halide perovskite FA_0.95_Cs_0.05_PbI_3_, suggesting an ultrafast transfer of spin information prior to its relaxation. The fluence dependence and distinct results for polycrystalline vs crystalline samples further characterize the mechanisms of spin dynamics in these important semiconductor systems.

## Results

Figure 1a,b shows SEM images of the FA_0.95_Cs_0.05_PbI_3_ samples. The average grain size in the lateral dimension of Figure 1a is approximately 700 nm. The crystal in Figure 1b has no evident defects over a mm-size area. Figure 1c shows a transmission spectrum of the polycrystalline thin film (the single crystal was too optically dense to measure an absorption spectrum). Two wavelengths were used to access different regimes of excess energy above the material band gap. Unless otherwise noted, data shown are for the 750 nm excitation and probe measurements, with additional measurements presented in the SI. The fluence regime presented corresponds to an excitation density of roughly 3 × 10^16^ cm^–3^ at the low end to 5 × 10^18^ cm^–3^ for the highest power measurements.

**Figure 1:**
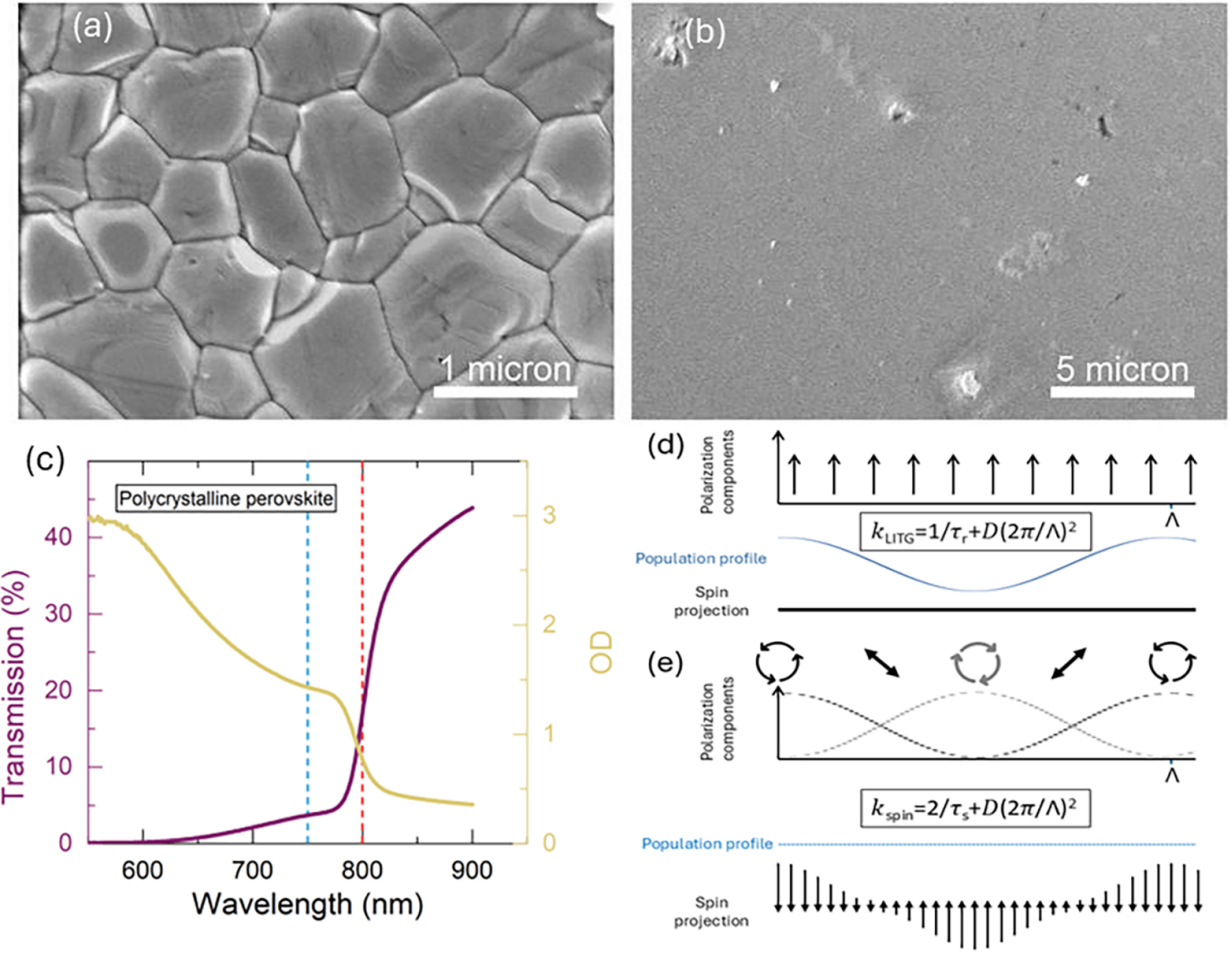
SEM images of (a) polycrystalline and (b) single crystal samples. (c) Transmission and absorption spectra of polycrystalline sample, with dashed lines at the positions of LITG pumping. Schematic of LITG experiment for (d) population and (e) spin gratings.

Figure 1d shows a schematic of the polarization along a grating period for the standard LITG experiment, where the beams are adjusted to crossed polarization for the spin diffusion measurement in Figure 1e. This is indicated by the circularly polarized icons that illustrate the distinct phase of the interacting orthogonal polarizations at varying positions on the sample. The primary distinguishing feature of LITG experiments is the pattern of population (or spin) amplitudes undergoing periodic modulation across the grating area that is effectively ‘written’ into the sample through interaction of the two noncollinear polarized light beams with the excitonic transition moments. The decay of this grating leads to the reduced amplitude of the delayed diffracted beam. This decay occurs through a combination of excited-state relaxation and motion of carriers or excitons from the peaks to the valleys of the grating. As shown in the equations in Figure 1d,e, these two contributions are distinguished by changing the angle of the incident beams, which alters the grating period. Excited-state relaxation is independent of the period, while the diffusive component slows as the grating period elongates.

Figure 2 shows LITG transients recorded at a fixed grating period Λ = 14.4 μm at different excitation fluences, for a polycrystalline perovskite layer (a) and a single crystal (b). Both samples present faster decays with the increasing excitation energy density due to the input from higher-order recombination mechanisms. Meanwhile, the decay rates appear to be faster in a polycrystalline layer, when comparing similar excitation fluences.

**Figure 2:**
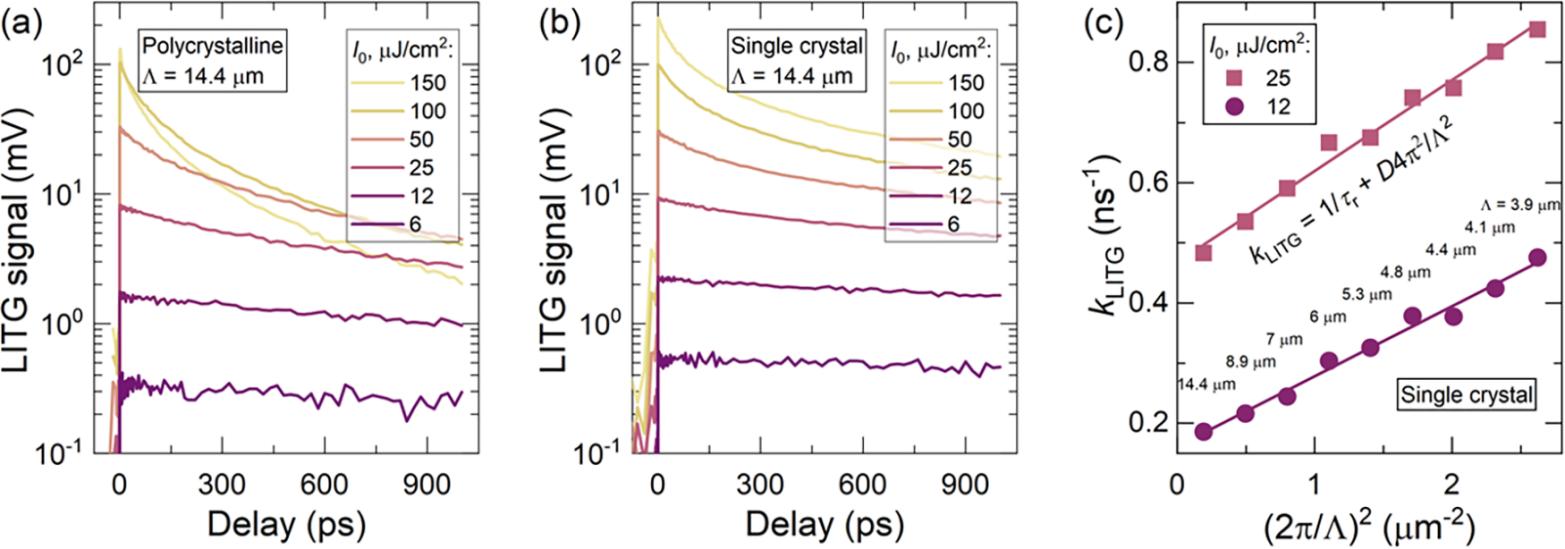
Raw LITG transients for (a) polycrystalline and (b) single crystal samples, measured at a fixed grating period of 14.4 μm, at different excitation fluences, indicated in the insets. (c) LITG decay rate constant plotted versus the inverse square of grating period for a single crystal sample to determine the diffusion coefficient via the slope, at two different excitation fluences of 12 μJ/cm^2^ (circles) and 25 μJ/cm^2^ (squares).

In Figure S1, LITG transients for a single crystal are recorded at a fixed excitation fluence of 25 μJ/cm^2^, for different induced grating periods as indicated. The increasing decay rate with smaller Λ signifies the presence of carrier diffusion. The decay time constants τ_g_, used for nonequilibrium carrier diffusivity calculations, were evaluated by fitting the transients within a 2–300 ps window (fitting example for a 3.9 μm transient is shown in the inset of Figure S1). Figure 2c shows the decay rate constant (*k*_LITG_ = 1/τ_g_) plotted over the inverse grating period squared, at excitation fluences of 25 μJ/cm^2^ and 12 μJ/cm^2^. The symbols depict *k*_LITG_ values for different Λ, while solid lines represent linear data fits.

The ambipolar carrier diffusion coefficient *D* and carrier lifetime τ_r_ dependencies on excitation fluence are shown in Figure 3a,b. It is seen that for both samples the nonequilibrium carrier diffusivity increases with higher excitation, which is likely a collective effect of carrier delocalization and degeneracy. However, the single crystal shows significantly higher diffusivity values at lower excitations (<50 μJ/cm^2^), implying weaker carrier localization and band-like diffusion regime.^20^ The carrier lifetimes drop with excitation in both samples with a similar trend, at least in the ≥12 μJ/cm^2^ range, showing similar rates of higher-order recombination mechanisms. However, the lifetime at low carrier densities is longer in a single-crystal perovskite, implying much slower Shockley-Read-Hall nonradiative recombination due to a lower density of electrically active defects. Very likely the more active nonradiative recombination in polycrystalline sample is caused by grain boundaries, which were shown to act as efficient nonradiative recombination centers.^29,30^ The square root of the diffusion coefficient and carrier lifetime product – the diffusion length ( $L_{D}=\sqrt{D\tau_{r}}$ ) – is also higher in a single crystal (Figure 3(c)), exceeding 1 μm at lowest excitations. To confirm that the plateau of diffusion lengths has been reached by roughly 3 μJ/cm^2^, we performed additional experiments at lower fluence (using 800 nm excitation). Despite lower signal, the values indeed have reached saturation, and the full set measured *D*, τ_r_, and *L*_D_ are summarized in Tables S1 and S2. We note that the diffusion lengths found here for a single crystal are similar to other measurements made at room temperature.^30,31^ Further, the ‘pinned’ diffusion length for the polycrystalline film (∼380 nm) is roughly half the average grain size (∼700 nm), meeting with expectations for grain boundaries representing either electron sinks due to active surface-like recombination^32^ or efficient scattering interfaces.^33^

**Figure 3:**
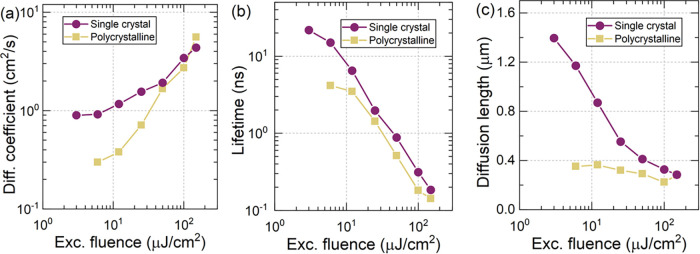
(a) Carrier diffusion coefficient, (b) lifetime, and (c) diffusion length for polycrystalline (yellow squares) and single crystal (purple circles) samples at different excitation energy fluences.

For the spin diffusion coefficient determination, two pump beams with orthogonal linear polarizations were used. In such a case, no light intensity modulation occurs, but the light polarization is modulated with the period identical to that of a regular carrier grating, produced by pump beams with parallel linear polarizations. A linearly polarized probe can be diffracted on the grating, because both its right and left circular components interact with the two periodically occurring circular components of the grating. As a result of this interaction, the polarization of the diffracted probe beam is orthogonal to that of the incident probe, therefore a wire-grid polarizer was fitted in front of the diffracted beam detector to pass-through only the horizontally polarized pulses.

The spin relaxation LITG transients in a polycrystalline layer and a single crystal at 60 μJ/cm^2^ excitation fluence are shown in Figure 4(a) and (b), respectively. The signals decay on a picosecond order, similarly to the pump–probe spin relaxation transients (Figure S2), which is several orders faster than for standard LITG measurements (Figure 2) that monitor ambipolar carrier transport. Since the differences between transients were minute (purple curves represent largest, yellow – smallest induced grating periods), a large number of gratings was used for statistical reasons. The single crystal was stable and did not degrade even after full-day measurements at a single spot, therefore as many as 96 transients were measured for a single excitation fluence. The polycrystalline layer, on the other hand, was prone to degradation, therefore the number of transients was limited to 30 per spot, with each excitation fluence measured at a unique location.

**Figure 4:**
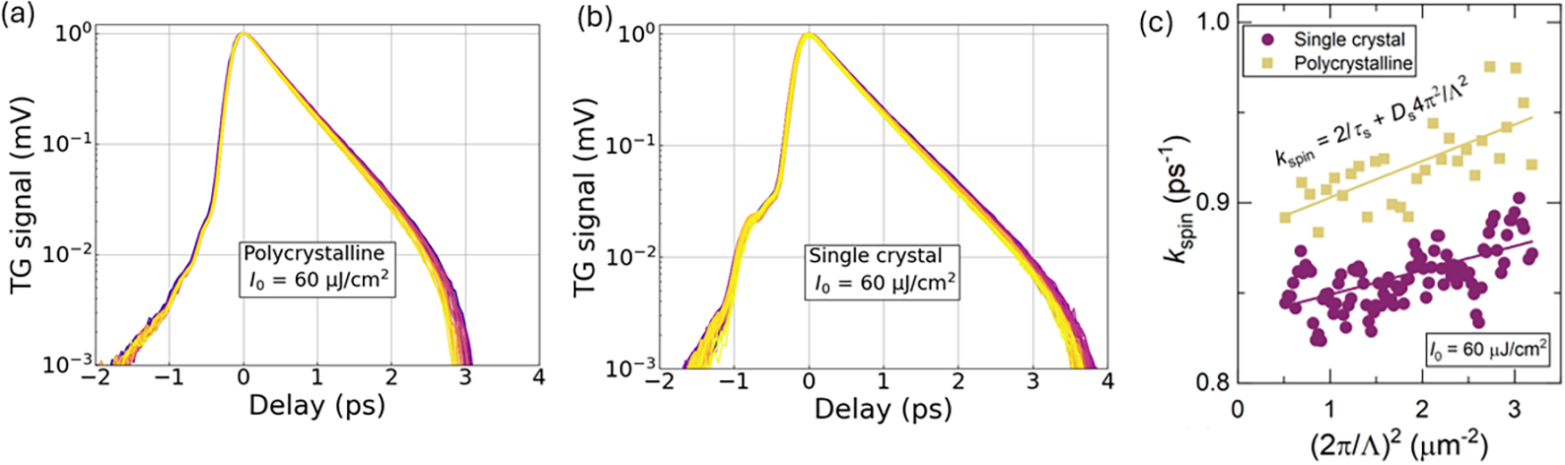
Cross-polarized LITG transients for various grating spacings for (a) polycrystalline and (b) single crystal samples, at a fixed excitation fluence of 60 μJ/cm^2^. (c) Spin grating decay rate constant plotted versus the inverse square of grating period for a pollycrystalline (yellow squares) and single crystal (purple circles) samples to determine spin diffusion coefficient for (a) and (b) measurement sets.

Figure 4c shows the decay rate constants plotted over the inverse grating period squared for cases depicted in Figure 4a,b. A steeper slope for a polycrystalline layer indicates higher spin diffusivity values, which holds for the entire excitation fluence range, as seen in Figure 5a. Here, the values are a result of averaging over 4 measurement runs for a single crystal, and over 9 for a polycrystalline layer (measurements in the latter had larger standard deviation). The spin diffusivity is derived similarly to the population diffusivity, except that the grating decay τ_g_ now involves contributions from spin relaxation and excited-state relaxation, although we can safely ignore the latter based on experiments shown above. Spin diffusivity increases with excitation for both cases, exceeding 100 cm^2^/s values, which is two-orders of magnitude higher than for the ambipolar carrier diffusion (Figure 3a). Spin relaxation times, obtained from LITG measurements, are similar for both samples (Figure 5b, solid symbols) and drop from nearly 4 ps to under 2 ps with the increasing excitation fluence.

**Figure 5:**
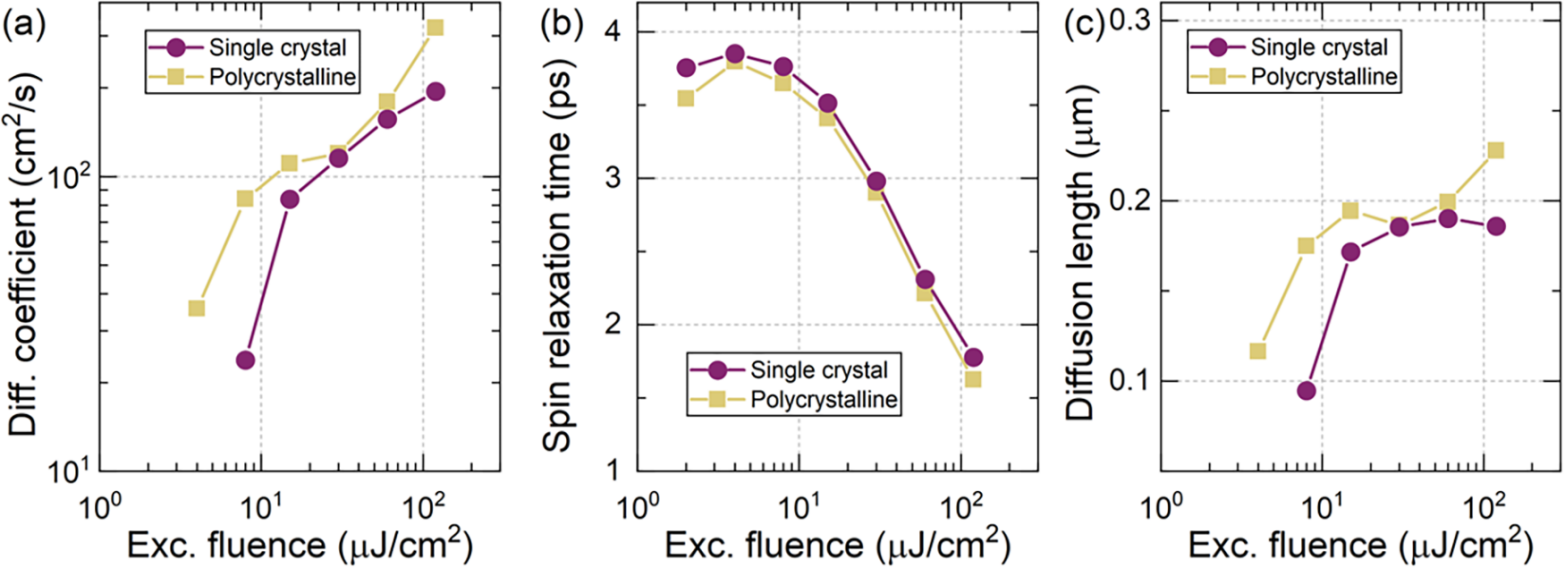
(a) Spin diffusion coefficient, (b) spin relaxation time, and (c) spin diffusion length for the polycrystalline (yellow squares) and single crystal (purple circles) samples at different excitation energy fluences.

Spin relaxation times, obtained from pump–probe (PP) measurements (open symbols, data shown in Figure S2), behave similarly with excitation as with LITG. The spin diffusion length follows closely with the diffusivity trend (Figure 5(c)) and reaches ∼0.19 μm at intermediate excitation fluences for both samples. Spin diffusivity (a), relaxation time (b), and diffusion length (c) values are summarized in Table S3. A presentation of uncertainty analysis is also found in the SI.

## Discussion

The spin properties of lead-halide perovskites have been extensively studied, uncovering a rich variety of spin physics and revealing Lande g-factors and spin relaxation/dephasing times, particularly at low temperature.^34–36^ Spin diffusion investigations in these materials encompass a much smaller footprint; however, a related study with a similar composition of crystalline perovskite material to what was used here was recently undertaken using MOKE spectroscopy in a microscopy mode.^37^ There, the sample temperature was estimated to be 25K, yielding a longer spin relaxation time (∼15 ps) than we observed at room temperature (∼3 ps). The dramatic lengthening of spin relaxation time with low temperature is well established.^3^ Photoexcited bulk perovskites at room temperature are reported to undergo ps-scale spin relaxation via the Elliott–Yafet and D’yakonov–Perel mechanisms, and we anticipate similar pathways are present here.^38^ Interestingly, the spin diffusion coefficient also rose significantly with increasing fluence and was found to be roughly 100 cm^2^/sec in a range that overlaps with our laser excitation conditions. However, a recent study of exciton and spin diffusion in a 2D perovskite using circularly polarized photoluminescence came to the opposite conclusion, that spin diffusion lagged behind exciton diffusion.^39^ We note that the diffusion coefficient in that case was determined through ns-scale and steady-state measurements, which naturally integrate over the time scales of geminate exciton exchange interactions and may reflect a distinct dynamical regime than accessed here.

An additional report about spin diffusion in 2D perovskites used Faraday rotation imaging on ultrafast time scales to implicate interexciton exchange interactions as the source of intensity-dependent spin diffusion that drives excitons with the same angular momentum projection quantum number away from each other in an ultrafast fashion.^11,40^ Like microscopy experiments with tightly focused beams, we produce regions of high spin-polarized exciton density with the cross-polarized LITG excitation, despite the relatively low average excitation density and the near-constant spatial distribution of overall population density. In the conventional LITG experiment, the population differences drive diffusion, but there is no additional repulsive force because all angular momentum projections are equally produced across the spatial population profile (the single linear polarization of the photons carries no angular momentum). Therefore, the transients would lack the exchange-driven ps-scale decay component associated with what is sometimes termed “superdiffusion” and found in various other semiconductors exhibiting spin-dependent transport.^41–43^

Layered systems are more natural environments for excitonic effects than bulk perovskites due to the enhanced exciton binding energy. However, the effects of exchange repulsion may be also found in a bulk 3D perovskite, which largely supports charge carriers at room temperature but with significant Coulomb interactions.^44,45^ We note the influence of composition and temperature on the role of excitons vs carriers in spin relaxation measurements.^46,47^ One other contributing factor is the resonant enhancement of third-order nonlinear polarization, which we previously showed tracks the excitonic position for bulk methylammonium lead halide perovskites at room temperature.^48^ It is an interesting question whether the loss of cross-polarized LITG signal actually represents the motion of excited states to different regions of the phase grating (e.g., monopolar diffusion of electrons further accelerated by exchange repulsion), or the interaction between proximal spins leads to spin flips mediated by exchange,^49^ without ultrafast excited state motion (i.e., a pure spin current). At room temperature, one might expect enhanced scattering with increased excitation density to hinder the ballistic nature of superdiffusive motion, leaving the pure spin transport mechanism as more likely to be measured here. Further investigation of this phenomenon is worthwhile.

We note another possible source of faster spin vs carrier diffusion, which is unbalanced electron and hole diffusion coefficients.^50^ Although ambipolar diffusion of carriers is limited by the slower of the diffusive species, spin diffusion can occur as quickly as the faster carrier moves, as long as the majority carrier doping exceeds a certain threshold such that the majority carrier population by itself represents a “sea” of up and down spins.^51^ The samples studied here are not intentionally doped but predicted to be at least lightly p-type,^52,53^ increasing in doping for air-exposed samples.^54^ This would suggest the slower minority carrier motion (electron) determines the diffusion coefficient. However, the faster-moving holes determine the spin diffusion coefficient, accelerating spin diffusion by roughly 1 order of magnitude with respect to carrier diffusion. This acceleration remains roughly 10-fold lower than the values we observe, suggesting that the exchange-mediated superdiffusion may also be playing a significant role, particularly at higher excitation density.

In contrast to the population diffusion results that show a large difference between polycrystalline and single crystal samples, spin diffusion is mostly unaffected by the sample crystal grain size. One might expect that as spin relaxation times increase at low temperature, the spin diffusion lengths will increase significantly, and then the influence of the grain boundaries on spin diffusion could be detected. Regardless, the ability to stimulate spin motion with a simple change of light polarization over a length scale greater than 100 nm and within a few ps at room temperature represents a unique opportunity to design opto-spintronic elements from tailorable materials.

## Conclusion

Light-induced transient grating (LITG) experiments performed on a mixed cesium/formamidinium lead iodide single crystal perovskite demonstrate exciton diffusion beyond 1.5 μm and spin diffusion up to about 200 nm at room temperature. Whereas excited-state diffusion coefficients are similar at about 1 cm^2^/sec for both polycrystalline and single crystal samples, diffusion lengths are much longer for single crystals. In contrast, spin diffusion coefficients are larger by almost 2 orders of magnitude, peaking above 100 cm^2^/sec for both single crystal and polycrystalline samples. We propose that the unique properties of cross-polarized LITG detect two distinct mechanisms that may drive the effective diffusive motion between oppositely oriented angular momentum projection regions of the phase grating on ultrafast time scales. Leveraging these interactions in light-induced spin transport architectures could lead to fast and efficient operations in spin-optical schemes for memory, logic, or other computing applications.

## Experimental Methods

### Light-Induced Transient Grating (LITG) Spectroscopy

Light-induced transient grating (LITG) experiments were performed using a HARPIA-TG spectrometer (Light Conversion) coupled to Yb-doped solid state femtosecond laser sources and optical parametric amplifiers (OPAs). Measurements were carried out using either a PHAROS (Light Conversion) laser system (250 fs, 6 W) with an ORPHEUS OPA (Light Conversion) operating at 30 kHz, or a CARBIDE (Light Conversion) laser system (200 fs, 40 W) coupled to an I-OPA (Light Conversion) operating at 100 kHz. We note that the automation enabled by the HARPIA-TG allowed for long-term measurements and many repeated scans to ensure proper statistics.

Unless stated otherwise, the pump pulse wavelength was set to 750 nm, while probing was carried out with the fundamental radiation (1030 nm). The probe beam diameter was ∼100 μm throughout all the experiments, meanwhile the pump beam diameter was ∼520 μm and ∼650 μm, for the carrier and spin dynamics measurements, respectively. All measurements were performed at room temperature in the transmission mode. All measurements were performed at room temperature in the transmission mode. Three different experiment geometries were employed to acquire parameters of both exciton and spin dynamics (see Figure 6). The temporal resolution is approximately 280 fs.

**Figure 6:**
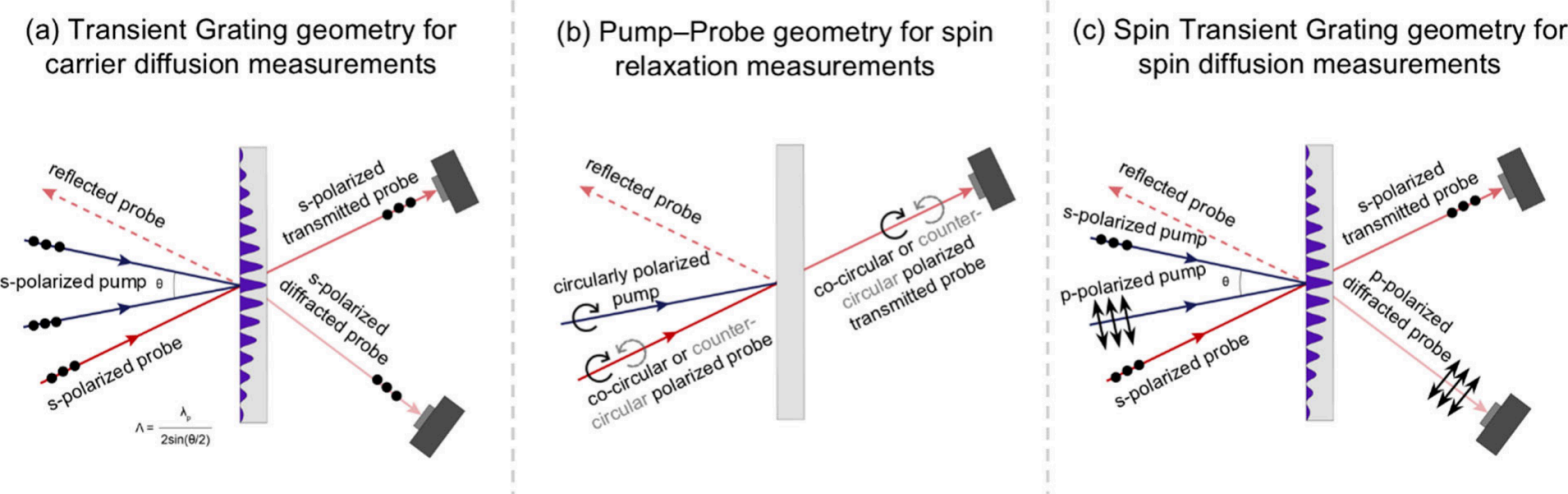
Schematic representation three different experimental (Harpia-TG) setups for probing charge carrier and spin dynamics.

#### a Transient Grating Geometry for Carrier Diffusion Measurements (Figure 6a)

In a transient grating geometry for charge carrier diffusion measurements, two s-polarized pump pulses overlap both spatially and temporally at the sample plane (Figure 1a). The interference field of these pump pulses causes the local excitation of charge carriers with the same pattern, resulting in a so-called transient excitation population grating. The period of this grating depends on an angle between two pump pulses (θ) as well as on a pump wavelength (λ_p_), following:

$$\Lambda =\frac{\lambda_{p}}{2\sin\left(\frac{\theta }{2}\right)}$$

The time-delayed s-polarized probe beam is then transmitted through the sample or diffracted from the created transient grating. The diffracted beam decay is measured at various grating periods, and each decay transient is fitted with a single-exponential function. The retrieved reciprocal decay constants (τ_g_^–1^) are plotted as a function of $({\frac{2\pi }{\Lambda })}^{2}$ .

As the laser-induced grating decays due to carrier recombination (with the rate of $\frac{1}{\tau_{r}}$ ) and carrier diffusion (with the rate of $\frac{1}{\tau_{D}}$ ):

$$\frac{1}{\tau_{g}}=k_{\mathrm{LITG}}=\frac{1}{\tau_{r}}+\frac{1}{\tau_{D}}=\frac{1}{\tau_{r}}+\frac{4\pi^{2}D}{\Lambda^{2}}$$

The tangent of the plotted function’s linear fit provides the carrier diffusion coefficient *D*, while the intercept at ${\left(\frac{2\pi }{\Lambda }\right)}^{2}=0$ provides the intrinsic carrier recombination rate $\frac{1}{\tau_{r}}$ . A more in-depth descriptions of the method can be found in refs 55 and 56. The employed HARPIA-TG spectrometer allows the predefined continuous variation of angles between two pump pulses, thus allowing faster recording of light-induced transient grating kinetics and different transient grating periods. In the spectrometer, the pump beam first passes through a static beam splitter (a Wollaston prism or a laser-etched grating). The resulting beam pair is collimated by a parabolic mirror, ensuring a constant distance between the two beams, and then is passed through a system comprised of two prisms – one fixed and one mounted on a translational stage. By translating the second prism, the distance between the parallel beams can be continuously varied. Focusing such a variation with a second parabolic mirror results in a continuously tunable angle between the beams and, thus, the grating period.

#### b Pump–Probe Geometry for Spin Relaxation Measurements (Figure 6b)

The spin relaxation time values were measured using the same HARPIA-TG setup. In this case, a conventional pump–probe configuration was employed by blocking one of the pump beams. The remaining pump beam was circularly polarized by introducing a quarter-wave plate. The time-delayed probe beam was set to be either cocircularly or counter-circularly polarized with respect to the pump beam also by employing a quarter-wave plate. To obtain the spin decays, transients with counter-circular polarizations were subtracted from those with cocircular ones, with the spin relaxation rate being 2/τ_s_.^57^

#### c Spin Transient Grating Geometry for Spin Diffusion Measurements (Figure 6c)

For the spin diffusion coefficient determination, two pump beams with orthogonal linear (s- and p-) polarizations were used. A wire-grid polarizer was placed in front of the detector to transmit only the horizontally polarized diffracted probe pulses. The spin diffusion coefficients were obtained following the same procedure as described in Section (a), with a slight modification to the spin relaxation rate term:^58^

$$k_{\mathrm{spin}}=\frac{2}{\tau_{s}}+\frac{1}{\tau_{D}}=\frac{2}{\tau_{s}}+\frac{4\pi^{2}D}{\Lambda^{2}}$$

### Sample Preparation and Characterization

#### FA_0.95_Cs_0.05_PbI_3_ Polycrystalline Thin Film

A 2.2 M FAPbI_3_ stock solution in 2-methoxyethanol (2-ME) was prepared by dissolving 378 mg of FAI and 1014 mg of PbI_2_ in 1 mL of 2-ME at room temperature inside an N_2_ filled glovebox. A 1.8 M CsPbI_3_ stock solution in DMSO was prepared by dissolving 467 mg of CsI and 829.8 mg of PbI_2_ in 1 mL of DMSO. The CsPbI_3_ (DMSO) solution was stored at 45 °C and continuously stirred to ensure complete dissolution. A MACl solution (50 mg/mL) was prepared by dissolving MACl in 2-ME at room temperature. The final perovskite precursor was prepared by mixing 71.3 μL of 2.2 M FAPbI_3_, 4.6 μL of 1.8 M CsPbI_3_, 2.9 μL of DMSO, 22.3 μL of MACl (50 mg mL^–1^), and 48.9 μL of 2-ME.

#### FA_0.95_Cs_0.05_PbI_3_ Single Crystal

1.6 M Cs_0.05_FA_0.95_PbI_3_ precursor solution was prepared by dissolving CsI, PbI_2_, FAI in GBL in stoichiometric ratio and stirred at 60 °C for overnight. For crystal growth, the precursor solution was introduced between two facing MeO-2PACz coated ITO substrates to form a confined space.^59^ The temperature was increased from 60 to 110 °C at a rate of 3 °C h^–1^ to promote crystal nucleation and growth. The substrates were then separated and cooled to room temperature to obtain the single crystals.

SEM measurements were performed using a Hitachi S-4800 field-emission microscope at an accelerating voltage of 3 kV and a beam current of 7 nA. The perovskite films were imaged directly without applying any conductive coating.

## Supplementary Material



## Notes

Full LITG transients for the single crystal, tabulated diffusion parameters and relaxation rates vs fluence, pump–probe data, rate constant uncertainty analysis (PDF)
