# Supplementary material for: Charge Carrier and Spin Diffusion in a Polycrystalline and Single Crystal Lead Halide Perovskite Semiconductor
Source: ACS Photonics. 2026 Jun 29;13(14):3942–50. doi: 10.1021/acsphotonics.6c00657 (PMC13377605; doi:10.1021/acsphotonics.6c00657)
Supplement: Supplementary file 1 [file ph6c00657_si_001.pdf]

Supporting information for

Charge Carrier and Spin Diffusion in a Polycrystalline and Single Crystal Lead Halide Perovskite Semiconductor

Kazimieras Nomeika,[1,2] Justina Jovaišaitė,[1] Ramūnas Aleksiejūnas,[2] Xinwen Zhang,[3] Duong Nguyen Minh,[3] Md Azimul Haque,[3,4] Matthew C. Beard,[3,4] Joseph M. Luther,[3,4]* Justin C. Johnson[3,4]*

1. Light Conversion Ltd., Keramikų str. 2B, LT-10233 Vilnius, Lithuania
2. Vilnius University, Faculty of Physics, Institute of Photonics and Nanotechnology, Saulėtekis Ave. 3, LT-10257 Vilnius, Lithuania
3. National Laboratory of the Rockies, 15013 Denver West Pkwy, Golden, CO 8040, USA
4. RASEI, A Joint CU-Boulder/NLR Energy Institute, Boulder, CO 80309, USA

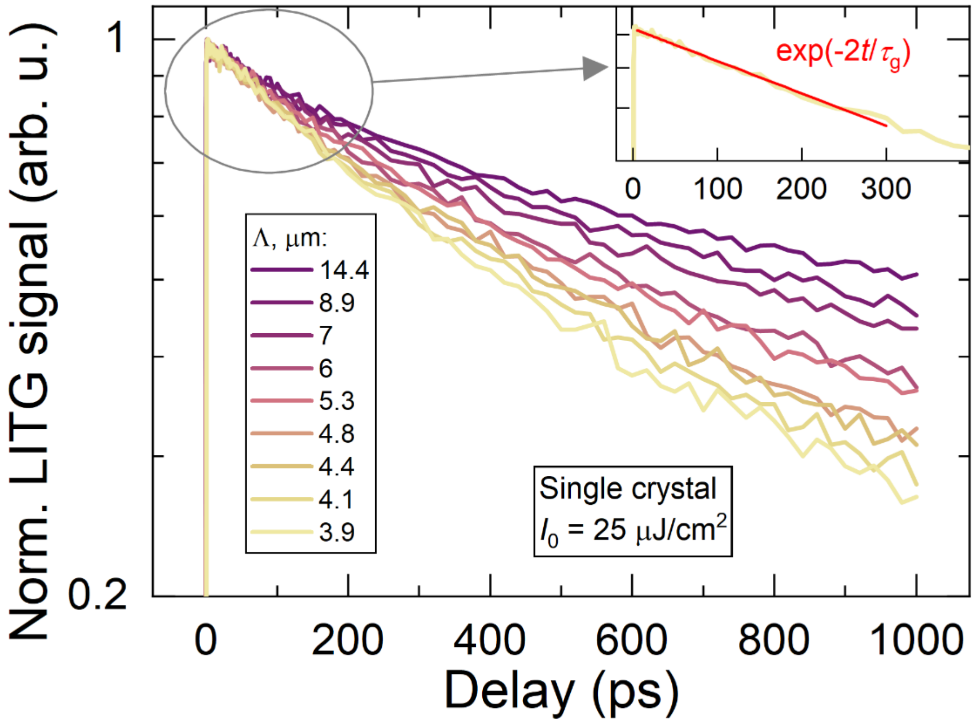

Figure S1. LITG transients for a single crystal, recorded at a fixed excitation fluence of 25 µJ/cm^2, for different induced grating periods. An example of LITG transient fitting is shown in the inset.

Table S1. LITG results for a range of excitation fluences for a single crystal acquired with the 800 nm pump.

I_{exc}, μJ/cm^2	D, cm^2/s	τ_r, ns	L_D, μm
48	1.57	5.25	0.91
25	1.28	9.24	1.09
17	1.27	10.57	1.16
12	1.25	14.40	1.34
8.6	1.07	14.39	1.24
6	0.97	15.18	1.49
5.1	1.06	14.12	1.22
4.3	1.34	22.03	1.72
3.6	1.13	13.90	1.25
3	1.19	30.33	1.90
2.5	0.91	8.83	0.89

Table S2. LITG results for a range of excitation fluences for a polycrystalline layer acquired with the 800 nm pump.

I_{exc}, μJ/cm^2	D, cm^2/s	τ_r, ns	L_D, μm
50	0.50	4.46	0.35
35	0.68	3.69	0.50
25	0.43	5.08	0.47
18	0.59	6.50	0.62
15	0.30	6.41	0.44

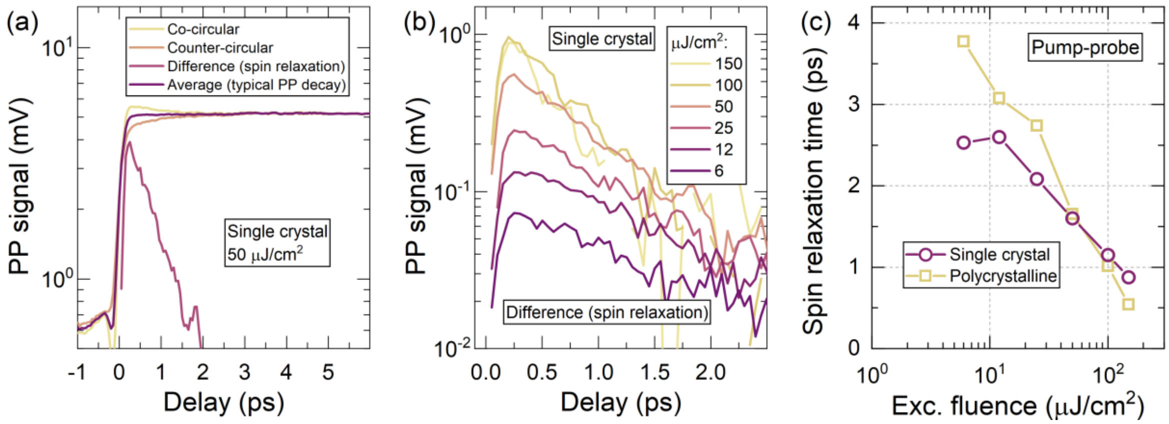

Figure S2. (a) Pump-probe transients in a single crystal, measured with co- and counter-circular polarizations, their average ($(I_{co} + I_{counter})/2$, typical PP decay) and difference ($(I_{co} - I_{counter})/2$, spin relaxation). The latter has been multiplied by 7 for an easier comparison. (b) Spin relaxation transients at different excitation fluences in a single crystal. (c) Pump-probe spin relaxation time dependence on excitation fluence.

Table S3. Spin dynamics data. We Note that even though diffusivity was not extracted at lowest excitation fluences as the differences between decay rates at various grating periods were indistinguishable, spin relaxation times were acquired by averaging.

	Polycrystalline			Single		
I_{exc}, μJ/cm^2	D, cm^2/s	τ_r, ns	L_D, μm	D, cm^2/s	τ_r, ns	L_D, μm
120	319.5	1.62	0.23	194.7	1.78	0.19
60	179.3	2.21	0.20	156.8	2.31	0.19
30	120.0	2.90	0.19	115.5	2.98	0.19
15	110.9	3.41	0.19	83.9	3.51	0.17
8	84.0	3.65	0.18	23.9	3.76	0.09
4	35.6	3.80	0.12		3.85	
2		3.54			3.75	

Uncertainty analysis of diffusion coefficients:

For the carrier-diffusion measurements, transients recorded at 9 grating periods for each excitation fluence were sufficient to extract diffusion coefficients with relative uncertainties

generally below 20%. In contrast, for the spin-diffusion measurements, we recorded up to 96 transients for the single-crystal perovskite at a given excitation fluence. Multiple independent measurement sets were also performed to assess reproducibility. These runs were acquired at different sample positions and, in some cases, on different physical crystals prepared on separate substrates. Thus, the uncertainty reported for the spin diffusion coefficient includes both the statistical uncertainty of the individual linear fits and the run-to-run reproducibility.

For example, for the single-crystal perovskite at an excitation fluence of 60 μJ cm^{-2}, the weighted-average spin diffusion coefficient obtained from multiple independent runs is **157 ± 11 cm^2 s^{-1}**. For comparison, the carrier diffusion coefficient measured at a similar excitation fluence of 50 μJ cm^{-2} is **1.9 ± 0.3 cm^2 s^{-1}**. For the individual single-crystal data set shown in main text Figures 4b and 4c, the extracted spin diffusion coefficient is **134 ± 18 cm^2 s^{-1}**. Therefore, even for a single representative run, the slope is statistically resolvable despite the modest variation in *k*spin with grating period. The agreement between this individual-run value and the weighted average obtained from multiple independent runs supports the robustness of the extracted spin diffusivity.

The uncertainty in the spin diffusion length was propagated from the uncertainties in both the spin diffusion coefficient and the spin relaxation time. At 60 μJ cm^{-2} for the single crystal, the spin relaxation time is **2.31 ± 0.07 ps**, giving a spin diffusion length of **0.19 ± 0.008 μm**. Thus, although the raw spin-grating kinetics show only weak grating-period dependence, the use of many grating periods, repeated transients, and independent measurement runs provides sufficient statistical confidence in the extracted spin diffusion coefficients and diffusion lengths.
